# Obstructive jaundice secondary to pancreatic head adenocarcinoma in a young teenage boy: a case report

**DOI:** 10.1186/1752-1947-5-439

**Published:** 2011-09-06

**Authors:** Sami Aziz Brahmi, Mohammed Khattab, Omar El Mesbahi

**Affiliations:** 1 Medical Oncology unit, Hassan II University Hospital, Fez, Morocco; 2 Department of Pediatric Oncology, Avicenne Hospital, Rabat, Morocco

## Abstract

**Introduction:**

Pancreatic adenocarcinoma is extremely rare in childhood. We report a case of metastatic pancreatic adenocarcinoma in a 13-year-old boy, revealed by jaundice.

**Case presentation:**

A 13-year-old Moroccan boy was admitted with obstructive jaundice to the children's Hospital of Rabat, Department of Pediatric Oncology. Laboratory study results showed a high level of total and conjugated bilirubin. Computerized tomography of the abdomen showed a dilatation of the intra-hepatic and extra-hepatic bile ducts with a tissular heterogeneous tumor of the head of the pancreas and five hepatic lesions. Biopsy of a liver lesion was performed, and a histopathological examination of the sample confirmed the diagnosis of metastatic ductal adenocarcinoma of the pancreas. Our patient underwent a palliative biliary derivation. After that, chemotherapy was administered (5-fluorouracil and epirubicin), however no significant response to treatment was noted and our patient died six months after diagnosis.

**Conclusion:**

Malignant pancreatic tumors, especially ductal carcinomas, are exceedingly rare in the pediatric age group and their clinical features and treatment usually go unappreciated by most pediatric oncologists and surgeons.

## Introduction

Beyond infancy obstructive jaundice is rarely encountered among children [1], and the pancreas is an extremely uncommon site of neoplasia in children and adolescents [2,3]. For this reason, our understanding of these tumors is still quite limited.

## Case presentation

**Figure 1:**
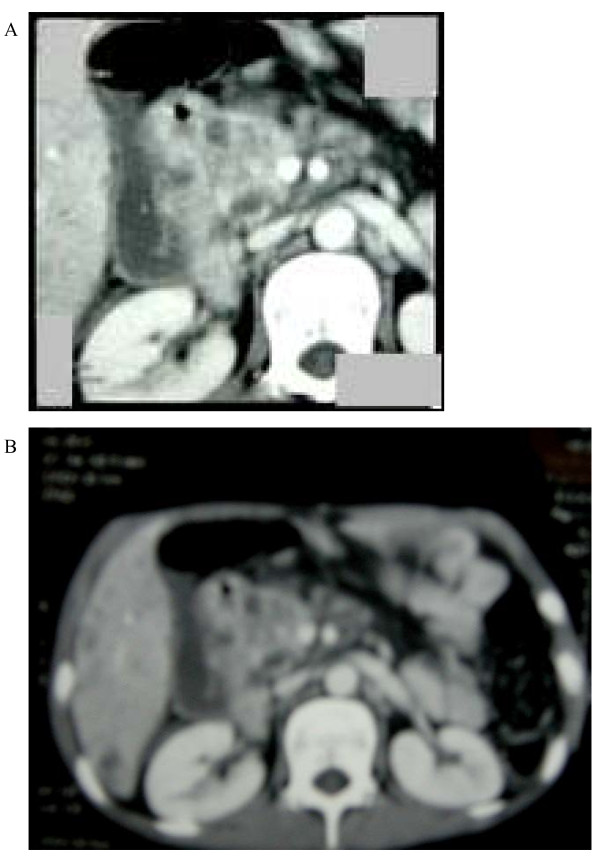
**Metastatic pancreatic adenocarcinoma in our 13-year-old patient**. A computerized tomography (CT) scan of the abdomen showed a tissular heterogeneous tumor of the head of the pancreas measuring 49 mm × 37 mm.

**Figure 2:**
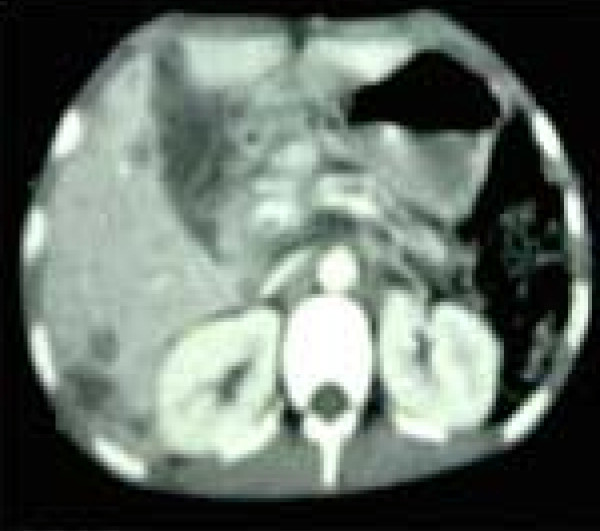
**Computerized tomography (CT) scan revealing metastatic lesions of the liver**.

A 13-year-old Moroccan boy was admitted with obstructive jaundice to the Children's Hospital of Rabat, Department of Pediatric Oncology. He had an illness of two months' duration that presented with jaundice, weight loss, melena and hematemesis. On physical examination, our patient had jaundice with hepatosplenomegaly. Laboratory study results showed a total bilirubin level of 26 mg/dL with a conjugated bilirubin of 18 mg/dL (normal level < 0.7 mg/dL), aspartate aminotransferase and alanine aminotransferase levels of 220 and 250IU/L, respectively (normal level < 45IU/L), and a hemoglobin level of 9 g/mL. Abdominal ultrasonography showed a dilatation of intra-hepatic and extra-hepatic bile ducts and hepatomegaly. Computerized tomography of the abdomen showed a dilatation of intra-hepatic and extra-hepatic bile ducts with a tissular heterogeneous tumor of the head of the pancreas measuring 49/37 mm (Figure 1A, B) and five metastatic lesions of the liver (Figure 2). Biopsy of one of the liver lesions was performed, and a histopathological examination of the sample confirmed the diagnosis of metastatic ductal adenocarcinoma of the pancreas. Our patient underwent a palliative biliary derivation. Chemotherapy was administered (5-fluorouracil and epirubicin); however, no significant response to treatment was seen and our patient died six months after diagnosis.

## Discussion

Pancreatic tumors in childhood most commonly present with a palpable mass or with pain [3]. Jaundice occurs much less commonly than in adults [3]. Tumors of the pancreas can arise from exocrine or endocrine cells. The following tumor types are described in children and adolescents: ductal adenocarcinoma, acinar cell carcinoma, pancreatoblastoma, solid pseudopapillary tumor, and pancreatic endocrine neoplasms (benign and malignant) [4]. In addition, there are reported cases of other tumor types, which either arise within the mass of the pancreas from other non-pancreatic cell types or immediately adjacent to it or involve the gland secondarily. Classic ductal adenocarcinoma of the pancreas, the common type in adults, is extremely rare in childhood. Most cases are in older literature. To examine the incidence trends and outcomes for children with pancreatic malignancies, the Surveillance, Epidemiology, and End Results Registry (1973 to 2004) was examined for pediatric patients with pancreatic malignancies (up to 19 years of age) [5]. Malignant pancreatic neoplasms were identified in 58 patients. Women outnumbered men 1.9:1 (38 versus 20) for an age population-adjusted incidence of 0.021 and 0.015 per 100,000. Ductal adenocarcinoma, was identified in seven patients. A 90-year (1918 to 2007), single institution, retrospective review of all patients with neoplastic pancreatic masses was performed and published recently [6]. Eighteen patients were identified with seven distinct histopathological subtypes. The most common were gastroenteropancreatic neuroendocrine, solid pseudopapillary, and acinar tumors. There were 6 benign and 12 malignant tumors. Surgery remains the keystone of treatment for pancreatic tumors in the pediatric age group, as in adults [7]. Long-term disease-free survival in childhood pancreatic malignancies is achievable with complete surgical resection [6]. There is no proven role for chemotherapy or radiation [4]. The information available in the literature, particularly with regard to the role of chemotherapy and radiation, is anecdotal [4]. These modalities should be reserved in unresectable and metastatic disease. Finally, it appears that ductal adenocarcinoma has a worse prognosis. According to a recent study, there was a significant difference in tumor type 15-year survival with ductal adenocarcinoma having the worst (23%) and solid cystic tumor the best (100%) [5].

## Conclusions

Malignant pancreatic tumors especially ductal carcinomas are exceedingly rare in the pediatric age group and their clinical features and treatment usually go unappreciated by most pediatric oncologists and surgeons.

## Consent

Written informed consent was obtained from the patient's next-of-kin for publication of this case report and any accompanying images. A copy of the written consent is available for review by the Editor-in-Chief of this journal.

## Competing interests

The authors declare that they have no competing interests.

## Authors' contributions

All authors have made significant contributions by making the diagnosis and through intellectual input in the case management and writing the manuscript.
